# Additive Manufacturing of Lignocellulosic Aerogels from Minimally Processed Waste Streams

**DOI:** 10.1002/smll.202412509

**Published:** 2025-07-24

**Authors:** Matteo Hirsch, Kosuke Ayama, Gert Preegel, Tiffany Abitbol

**Affiliations:** ^1^ Sustainable Materials Laboratory Institute of Materials École Polytechnique Fédérale de Lausanne Lausanne 1015 Switzerland; ^2^ Fibenol Tallinn 13522 Estonia

**Keywords:** additive manufacturing, cellulose nanomaterials, lignin nanoparticles, microcrystalline cellulose, thermal insulation

## Abstract

This study showcases the use of lignocellulosic pastes for the additive manufacturing of lightweight and load‐bearing porous materials. The pastes are obtained via a milder approach compared to the isolation of conventional cellulose nanomaterials and contain varying lignin contents depending on the extent of bleaching chemistry. The as‐produced pastes are compositionally and dimensionally heterogeneous, yet still exhibit excellent printability, enabling the facile fabrication of cellulose‐based materials through additive manufacturing. The rheological behavior of these pastes is investigated and their printability is assessed, demonstrating high resolution and shape retention. Upon freeze drying, the printed structures retain their 3‐D architecture without any appreciable shrinkage, a key performance indicator in additive manufacturing. Micro‐computed tomography is used to investigate the internal structure of the freeze‐dried structures, revealing a highly porous scaffold. These constructs possess mechanical strengths comparable to native cork and exhibit promising thermal insulation due to their high porosity. The direct 3D printing of crude lignocellulosic inks to obtain precisely architected structures, whose properties are defined but not limited by the heterogeneous nature of the inks, expands the applications of cellulosic inks in additive manufacturing.

## Graphical abstract

Lignocellulosic pastes derived from minimally processed wood waste are explored for 3D printing. These pastes demonstrate high printability and structural retention after freeze–drying. The resulting aerogels are lightweight, porous, and thermally insulating, with strength comparable to cork. This approach offers a sustainable alternative for additive manufacturing and insulation, with ongoing efforts to address scalability challenges.

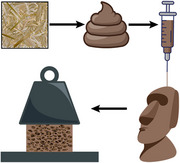

## 1 Introduction

Plastic pollution is a serious environmental threat.^[^ ^1^ ^]^ Each year, ≈70 million tons of plastic waste accumulate in the environment due to its non‐degradable nature.^[^ ^2^, ^3^, ^4^ ^]^ Efforts are underway to mitigate this crisis, with a considerable focus on recyclable plastics.^[^ ^5^, ^6^, ^7^, ^8^, ^9^ ^]^ Additionally, novel processing methods that support shorter and localized value chains offer promise in reducing the ecological footprint of plastic waste.^[^ ^10^, ^11^ ^]^ Within this framework, additive manufacturing emerges for its efficient use of resources, rapid prototyping capability, and ability for on‐demand production.^[^ ^12^, ^13^ ^]^

In the past decade, fast‐paced advances in additive manufacturing technologies have been paired with an exponential increase in ink design to adapt rheological properties with corresponding printing requirements.^[^ ^14^, ^15^, ^16^ ^]^ For example, current state‐of‐the‐art additive manufacturing of soft polymeric materials utilizes high viscosity polymeric solutions,^[^ ^17^, ^18^, ^19^ ^]^ synthetic and natural nanoparticles as rheological modifiers,^[^ ^20^, ^21^, ^22^, ^23^ ^]^ and granular precursors to optimize the viscoelastic properties of the ink,^[^ ^24^, ^25^, ^26^, ^27^ ^]^ thus improving overall printability and resolution.^[^ ^28^ ^]^

The widespread adoption of 3D printing technologies for rapid prototyping has, however, raised concerns over the toxicity and waste management associated with their associated materials.^[^ ^29^, ^30^ ^]^ As a result, the development of environmentally benign and sustainable alternatives for the replacement of conventional additive manufacturing materials is becoming increasingly important. For example, the use of bio‐sourced, renewable, and biodegradable precursors simplifies the end‐of‐life management of resulting materials, often reducing the overall environmental footprint of the process.^[^ ^31^, ^32^, ^33^ ^]^ Being intrinsically biodegradable and the most abundant biopolymer on Earth, cellulose has emerged as one of the most promising candidates for the replacement of synthetic precursors in additive manufacturing.^[^ ^34^, ^35^, ^36^, ^37^ ^]^ Recently, nanocellulose and lignin have been combined in a composite ink to 3D print wood‐like structures that demonstrate similar mechanical properties to that of balsa wood.^[^ ^38^ ^]^ Other examples include a lignocellulosic bioplastic with promising mechanical properties and UV and thermal stability fabricated from cellulose‐lignin slurries^[^ ^39^ ^]^ and the combination of wood flour, xyloglucan, and cellulose nanocrystals (CNCs) to fabricate wood‐like materials that mimic the chemistry, structural, mechanical, and thermal properties of timber.^[^ ^40^ ^]^ Pure cellulose aerogels have also been 3D printed with inks composed of CNCs and cellulose nanofibers (CNFs).^[^ ^41^ ^]^ Additionally, wood‐mimetic porous structures have been additively manufactured to desalinize water.^[^ ^42^ ^]^ However, these approaches rely on the use of highly processed and purified starting materials, thus making their environmental footprint still a concern. Furthermore, the extraction yield is low, and the resulting rheological properties are poor, such that they often require rheological modifiers to improve printability, making their scalability and production costs limiting factors for industrial use. Inks that employ minimally processed lignocellulosic waste streams that do not require any additives are still lacking.

Here, we demonstrate a novel approach to the additive manufacture of lignocellulosic pastes (LCPs) with varying lignin contents, where the initial crude LCP is obtained from the valorization of waste hardwood chips (Birch) based on a mild acid hydrolysis.^[^ ^43^ ^]^ Using this approach, lignin and hemicellulose are retained in the initial crude paste, and conventional bleaching chemistries can be applied to produce additional pastes further enriched in cellulose. The LCPs possess excellent rheological properties and printability, thus enabling the fabrication of arbitrarily complex 3D printed structures. Upon freeze‐drying, the lignocellulosic structure is converted into a free‐standing porous scaffold, referred to as a lignocellulosic aerogel (LCA). These printed aerogels exhibit remarkable mechanical and thermal insulating properties, thus making these materials a potential alternative to conventional insulating materials.

## 2 Results and Discussion

From a sustainability perspective, one‐pot methods for extracting lignocellulosic materials with high yield and multifunctional properties are highly appealing.^[^ ^44^ ^]^ Similarly, to reduce costs and the environmental footprint of cellulose‐based 3D printed structures, we utilize commercially produced LCPs that are obtained from a green extraction process, known as the Sunburst process.^[^ ^43^, ^45^ ^]^ This mild extraction favors the preservation of the natural compositional motif of wood, consisting of cellulose, hemicellulose, and lignin, thus enhancing extract yield and reducing waste. Significantly, the LCPs are produced at relatively high solids, above 17 wt.%, providing a rheological profile that is inherently suited for extrusion‐based additive manufacturing, and which can be further tailored depending on the amount of residual lignin in the LCP. Different from other cellulose‐based inks used in additive manufacturing, often formulated in a bottom‐up manner from distinct purified lignocellulose elements and additives,^[^ ^38^, ^46^, ^47^ ^]^ the LCPs described in this work are produced in a top‐down approach and require no external compositional or processing inputs, such as thickeners, binders, or fibrillation processes, to achieve the requirements of printability.

**Figure 1:**
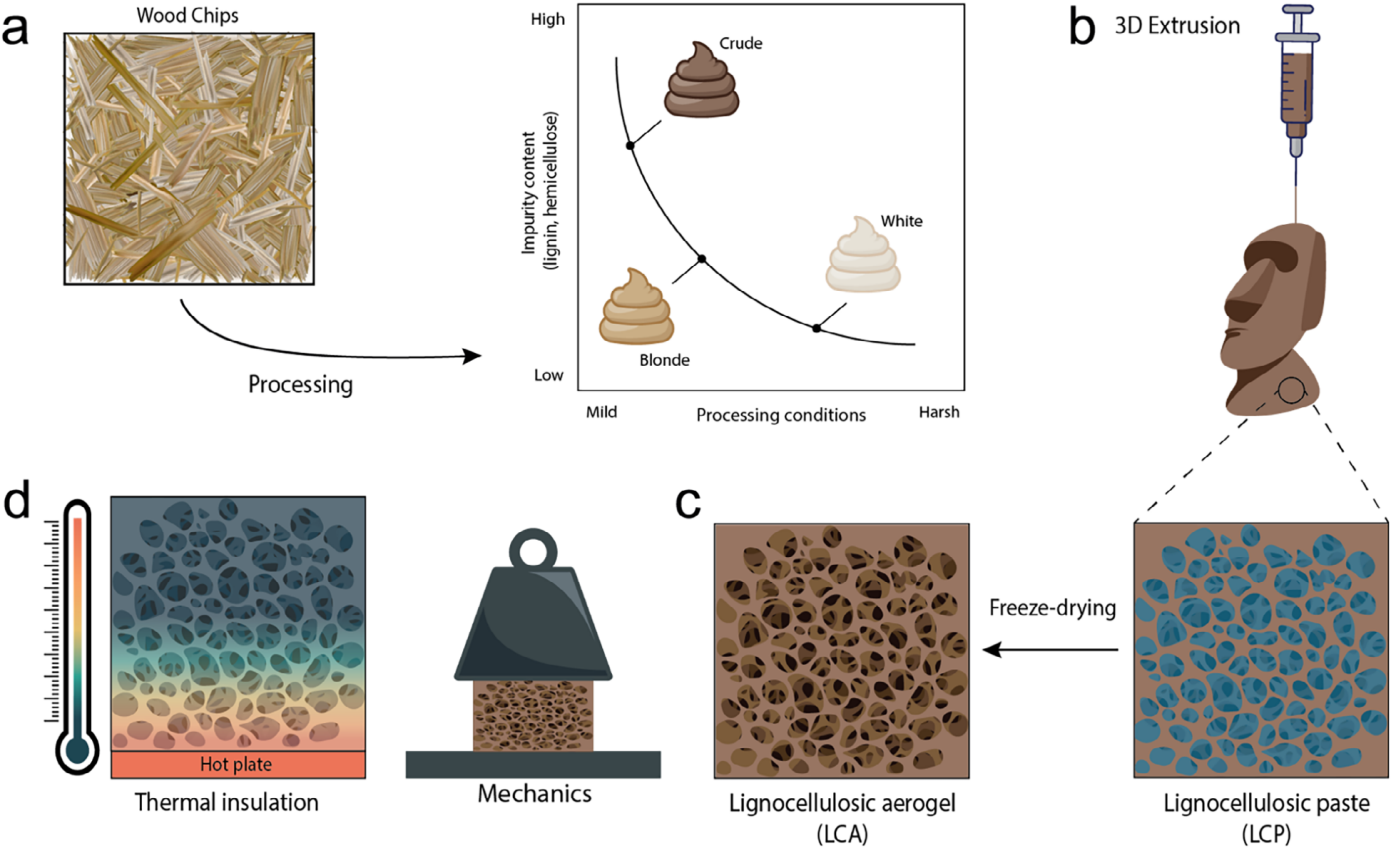
Schematic representation of the fabrication of 3D printed lignocellulosic aerogels and their properties and applications. a) LCP composition as a function of the degree of processing. b) Additive manufacturing of LCPs into arbitrarily complex shapes. c) Conversion of LCPs into LCAs by freeze‐drying. d) Applications of LCAs as thermal insulating and load bearing materials.

Based on their lignin content, the obtained LCPs are referred to as crude (LCP‐C), blonde (LCP‐B), and white (LCP‐W), as schematically shown in **Figure** **1**a, where LCP‐C is unbleached and LCB‐B/W have undergone different degrees of bleaching. The compositional analysis of the different LCPs is reported in Figure S1 (Supporting Information). We hypothesize that it is the unique combination of microcrystalline and nanocrystalline cellulose components, together with lignin nanoparticles, which makes these LCPs extremely appealing for additive manufacturing, as schematically described in Figure 1b. Specifically, we hypothesize that the free‐standing and high shape‐retaining properties of the 3D printed hydrogel structures stem from the favorable rheological properties of cellulose nanomaterials. Additionally, LCPs can be freeze‐dried to remove water, preserving a 3D porous architecture, yielding free‐standing LCAs, as shown in Figure 1c. The resulting LCA is load‐bearing, highly porous, and lightweight, features that are interesting for applications as load‐bearing and thermally insulating materials, as schematically shown in Figure 1d.

**Figure 2:**
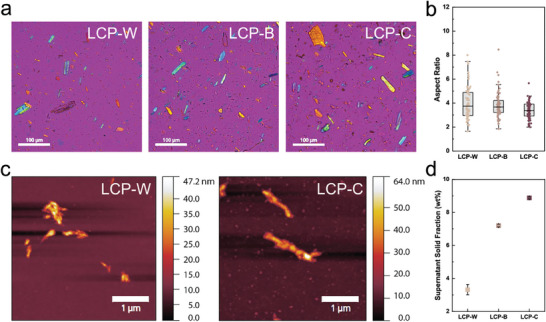
Characterization of LCPs. a) POM images highlighting the microcrystalline cellulose component of the different pastes at 0.1 wt.%. b) Aspect ratio of microcrystalline particles as a function of lignin content. c) AFM images of LCP‐W and LCP‐C nano‐fractions obtained by centrifugation of pastes at 0.01 wt.%. LCP‐C shows rod‐shaped aggregates and smaller nanoparticles attributed to lignin. d) Supernatant solid fraction as a function of lignin content.

To unravel the individual contributions within the heterogeneous LCP compositions, as well as to identify possible synergies, several analyses were conducted, including polarized optical microscopy (POM) of the micron‐sized components of the LCPs, as shown in **Figure** **2**a. The longitudinal (d_//_) and transversal (d⟂) axes of the birefringent cellulosic crystals reveal no significant differences across the three samples, as shown in Figure S2 (Supporting Information). Interestingly, a significant increase in aspect ratio (d_//_/d⟂) is observed with increasing degree of processing, as shown in Figure 2b. This behavior can be attributed to the more intensive extraction process that produces a higher degree of polydispersity of the microcrystals. However, the impact of lignin cannot be inferred from the light microscopy images, as lignin is usually present in the form of nanoparticles.

To account for the contribution from nanosized particles, atomic force microscopy was performed on the nano‐fraction component of LCP‐W, LCP‐B, and LCP‐C, as shown in Figure 2c and Figure S3 (Supporting Information). LCP‐W, LCP‐B, and LCP‐C show rod‐shaped CNC‐like aggregates of ≈1 µm in length, which are probably cellulosic in nature, whereas LCP‐B and LCP‐C also display smaller spherical features of ≈100 nm that are attributed to lignin nanoparticles.^[^ ^44^, ^48^ ^]^ Nano‐yield measurements were performed to quantify the colloidally stable fraction using a gravimetric approach to compare the solid contents obtained after centrifugation in the pellet and supernatant fractions. The supernatant solid content of LCP‐C is 3‐fold that of LCP‐W, as shown in Figure 2d. These results corroborate the presence of higher amounts of nanoparticles in LCP‐C compared to LCP‐B and LCP‐W.

**Figure 3:**
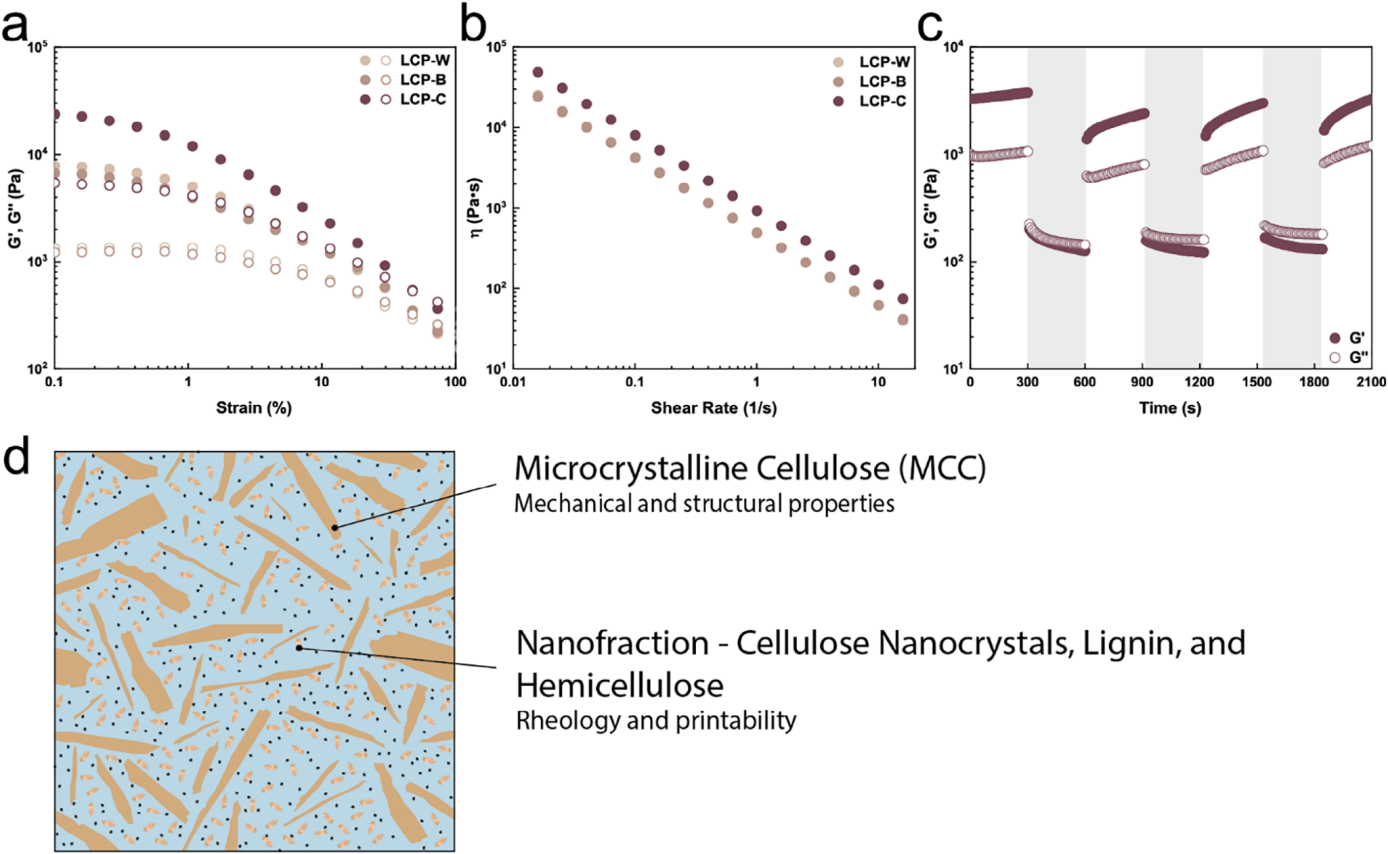
Rheological characterization of LCPs at 14 wt.% cellulose. a) Amplitude sweep of lignocellulosic pastes as a function of lignin content. Higher lignin content correlates to a higher G′, G″, and yield point. b) Viscosity measurement as a function of lignin content. All pastes display shear‐thinning behavior independent of lignin content. LCP‐B and LCP‐C overlap due to their similar rheological nature. c) Shear‐recovery measurement of LCP‐C. White areas correspond to a low shear strain regime (1%), while shaded areas correspond to a high shear strain regime (50%). The paste displays good shear‐recovery, confirmed by the rapid inversion of G′ and G″ upon release of the high shear stress. d) Schematic representation of the micro and nano‐composition of an LCP ink and their relative contribution to the rheological behavior.

A good ink for extrusion‐based 3D printing is shear‐thinning and possesses fast shear‐recovery, a storage modulus sufficiently high to retain its shape upon printing, and a yield point sufficiently low to ensure flawless extrusion of the material from the nozzle.^[^ ^15^, ^24^ ^]^ Additionally, the nano‐scale fraction of an ink is fundamental in imparting good rheological properties to the overall material.^[^ ^22^, ^23^ ^]^ To verify if the LCPs satisfy the rheological criteria for 3D printing, oscillatory rheology was performed on LCPs containing 14 wt.% of cellulose and different lignin contents. First, amplitude sweep measurements were carried out to evaluate the elastic response of the LCPs and their corresponding yield point, as shown in **Figure** **3**a and Figure S4 (Supporting Information). LCP‐C shows a more than 2‐fold increase in both storage (G′) and loss (G″) moduli compared to both LCP‐W and LCP‐B. Similarly, the yield point of the LCPs increases from ≈250 to 510 Pa with increasing lignin content. These increases are attributed to the increased amount of lignin present in LCP‐C (7 wt.%) compared to LCP‐W (0.5 wt.%). Second, frequency sweep measurements were performed to evaluate the shear‐thinning behavior of the LCPs, as shown in Figure 3b and Figure S5 (Supporting Information). Independent of the amount of lignin in the paste, all the LCPs display a shear‐thinning behavior where the viscosity decreases with increasing shear rate. Additionally, LCP‐C shows a higher overall viscosity attributed to the higher nano‐fraction present in this paste. Lastly, the ability of the LCPs to recover the stress upon shear release was evaluated by exposing the LCP‐C to alternating cycles of low shear strain (1%) and high shear strain (50%), as shown in Figure 3c. The paste displays a relatively fast shear stress recovery upon release of the high shear strain, suggesting good shape retention during 3D printing. An initial loss in recovery of ≈30% is observed between the first and second cycles, which is attributed to the anisotropic nature of microcrystalline cellulose particles that makes them susceptible to shear alignment.^[^ ^47^, ^49^ ^]^

To explore the contribution of the water to cellulose ratio on the rheology of the LCPs, and hence on their printability, rheological measurements were performed on LCP‐C containing 14, 12, and 10 wt.% cellulose, as shown in Figure S6 (Supporting Information). As expected, a decrease in solid content is paired with a gradual loss of viscoelastic and shear‐thinning properties caused by dilution of the paste. While LCPs containing 12 and 10 wt.% cellulose can still be 3D printed, the final construct will suffer from a lack of resolution and poor mechanical stability.^[^ ^15^, ^21^, ^22^, ^25^, ^50^, ^51^ ^]^ As a result, in the following sections, we focus on LCPs containing 14 wt.% cellulose. This may primarily be related to a threshold nano‐fraction concentration required in the pastes for printability.

In native wood, interactions between cellulose and lignin are mediated by the presence of hemicellulose. As our LCPs are extracted from wood through a mild hydrolysis, we speculate that the natural architecture is partially preserved in the final paste and is therefore responsible for its superior rheological properties. To evaluate if this assumption holds true, we first compare LCP‐W with and without hemicellulose, as shown in Figure S7 (Supporting Information). In the absence of hemicellulose, a 4‐fold decrease in G′ and G″ is observed, the viscosity of the paste is significantly reduced, as is the shear‐thinning behavior. Ultimately, to demonstrate that preserving the as‐extracted state of our LCP is also crucial, we compared the shear‐thinning behavior of our LCP‐C to a suspension of commercially available components mixed in the same proportions, as shown in Figure S8 (Supporting Information). By a simple tube inversion test, it is evident that a simple mixing of components is not enough to achieve an LCP with adequate rheological properties. However, we note that while the mixed compositions matched that of the LCPs in terms of bulk composition (14 wt.% cellulose, 8 wt.% lignin), they are unlikely to capture features related to morphology, size distribution, or solvent conditions. Nonetheless, we observe that the addition of commercial lignin to LCP‐W to match the LCP‐C composition results in very similar viscosities, further confirming the importance of the as‐extracted paste composition.

In summary, the rheology of LCPs can be assimilated to that of a composite ink system composed of a rigid microscopic particle and a soft, deformable nanofiller, as schematically shown in Figure 3d. The granular component, represented by MCC, contributes to the high shape‐retaining property of the ink, owing to the mechanical interlocking of individual microcrystals.^[^ ^27^ ^]^ The nanofiller component, composed of CNCs, lignin, and hemicellulose, acts as a rheological modifier, endowing the paste with shear‐thinning properties and making it 3D printable.^[^ ^21^, ^47^ ^]^

**Figure 4:**
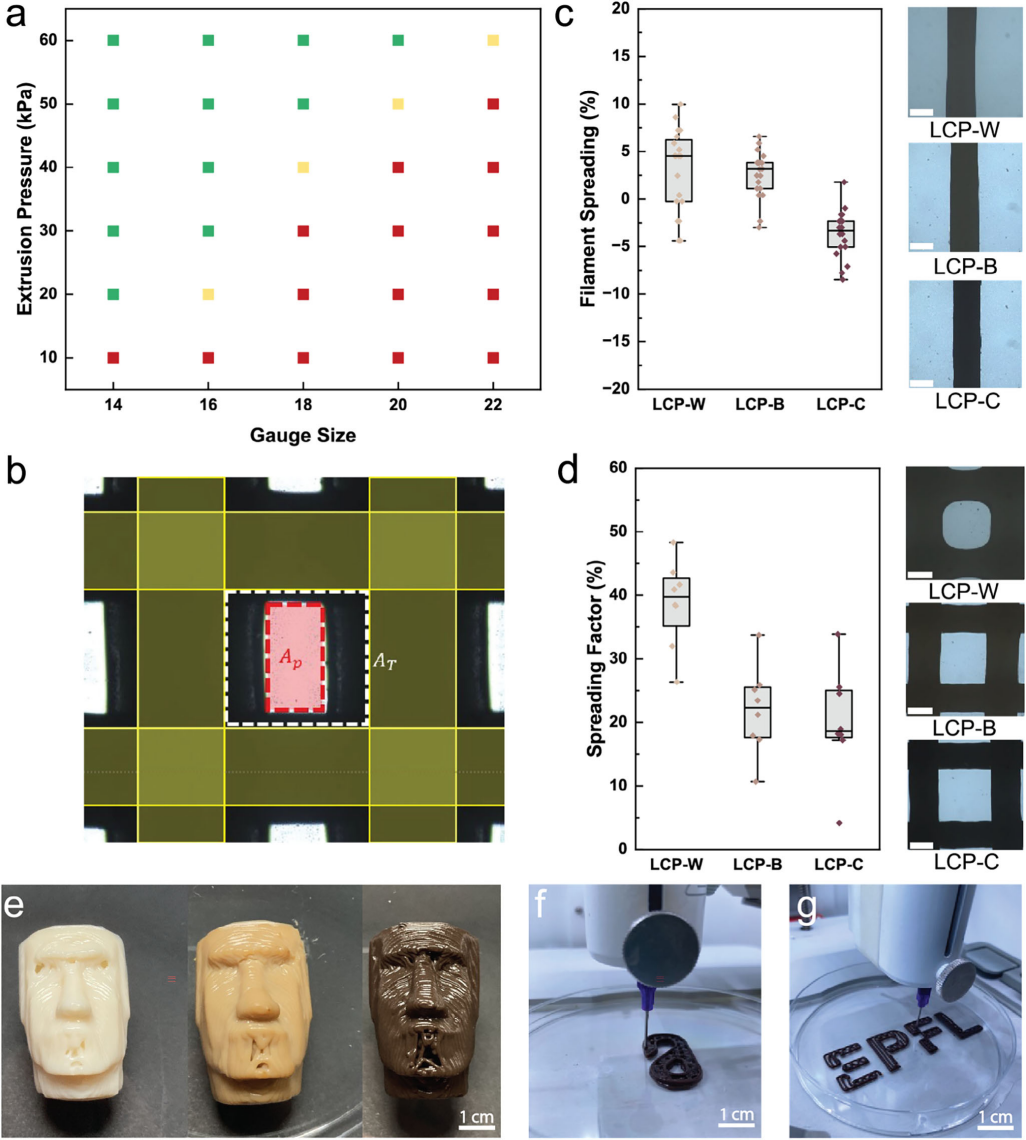
Printability characterization of LCPs. a) Printability window of LCP‐C as a function of gauge size and extrusion pressure. Red, yellow, and green data points represent no extrusion, partial extrusion, and continuous extrusion, respectively. b) Optical micrograph of an extruded grid element. Yellow shaded areas represent the nominal nozzle diameter, A_T_ represents the theoretical printed area, and A_P_ represents the effective printed area. c) Filament spreading assay as a function of LCP composition. Scale bar is 500 µm. d) Printing resolution assay as a function of LCP composition. Scale bar is 500 µm. e) Photographs of 3D printed Moai statues made with LCP‐W, LCP‐B, and LCP‐C. f) Photograph of a 3D printed LCP‐C hinge. g) Photograph of a 3D printed LCP‐C EPFL logo.

To ensure good printing resolution, the direct extrusion of a filament should occur continuously in the presence of an applied external pressure higher than the yield pressure of the ink. Additionally, the printed filament should retain the same nominal size of the nozzle throughout the entire printing process to prevent loss of resolution and structural collapse. To assess if this is the case for the LCP inks, a printability assay as a function of nozzle size, printing pressure, and paste composition was performed. First, the printing window of the LCPs was determined, as shown in **Figure** **4**a and Figure S9 (Supporting Information). As expected, smaller nozzle sizes (higher gauge numbers) require higher printing pressures to ensure correct extrusion. Furthermore, 5 mm s^−1^ was selected as the cutoff extrusion speed to differentiate the printing parameters at which a structure can be continuously fabricated, as shown in Movie S1 (Supporting Information). Interestingly, all LCPs display good extrudability at pressures below 60 kPa, thus suggesting overall good printability under low‐pressure conditions.

Once the suitable extrusion pressure was selected, the printing resolution was characterized to ensure the shape fidelity and stability of the final constructs. To evaluate the printing resolution of LCPs, the filament spreading factor was measured, providing the deviation of the filament diameter from the nominal nozzle size, as shown in Figure 4c. As observed, all the extruded filaments have a spreading factor below 20%, regardless of lignin content. Interestingly, LCP‐C filaments underwent a small contraction in the filament size, which might be related to the higher nano‐fraction in this ink, leading to more pronounced shear‐alignment in the extruded paste, and hence to a reduced final diameter.^[^ ^28^ ^]^ Furthermore, a good ink for 3D printing should retain its shape over time to avoid structural deformation and loss of resolution. To verify if the LCPs are suited for the additive manufacturing of complex shapes, the spreading factor of a 3D printed grid was studied, as shown in Figure 4b. Grids printed with LCP‐B and LCP‐C have a higher resolution with sharp edges and minimal reflow of the structure, as shown in Figure 4d. This behavior may be attributed to the higher concentrations of lignin in the paste that improve the shear recovery and prevent the reflow of the filaments after extrusion, as previously shown in Figure 3c. As a result, inks with the highest lignin contents, LCP‐B and LCP‐C, display spreading factors as low as 20% compared to values of ≈40% for LCP‐W, which contains the least lignin and the lowest nano‐fraction. Owing to the good printability and versatility of our LCPs, it is possible to additively manufacture arbitrarily complex structures that are free‐standing and display high shape‐fidelity. To demonstrate this feature, the same Moai statue structure was 3D printed with the different pastes, as shown in Figure 4e. Additionally, LCP‐C was used to print a hinge and the EPFL logo, as shown in Figure 4f,g, respectively.

**Figure 5:**
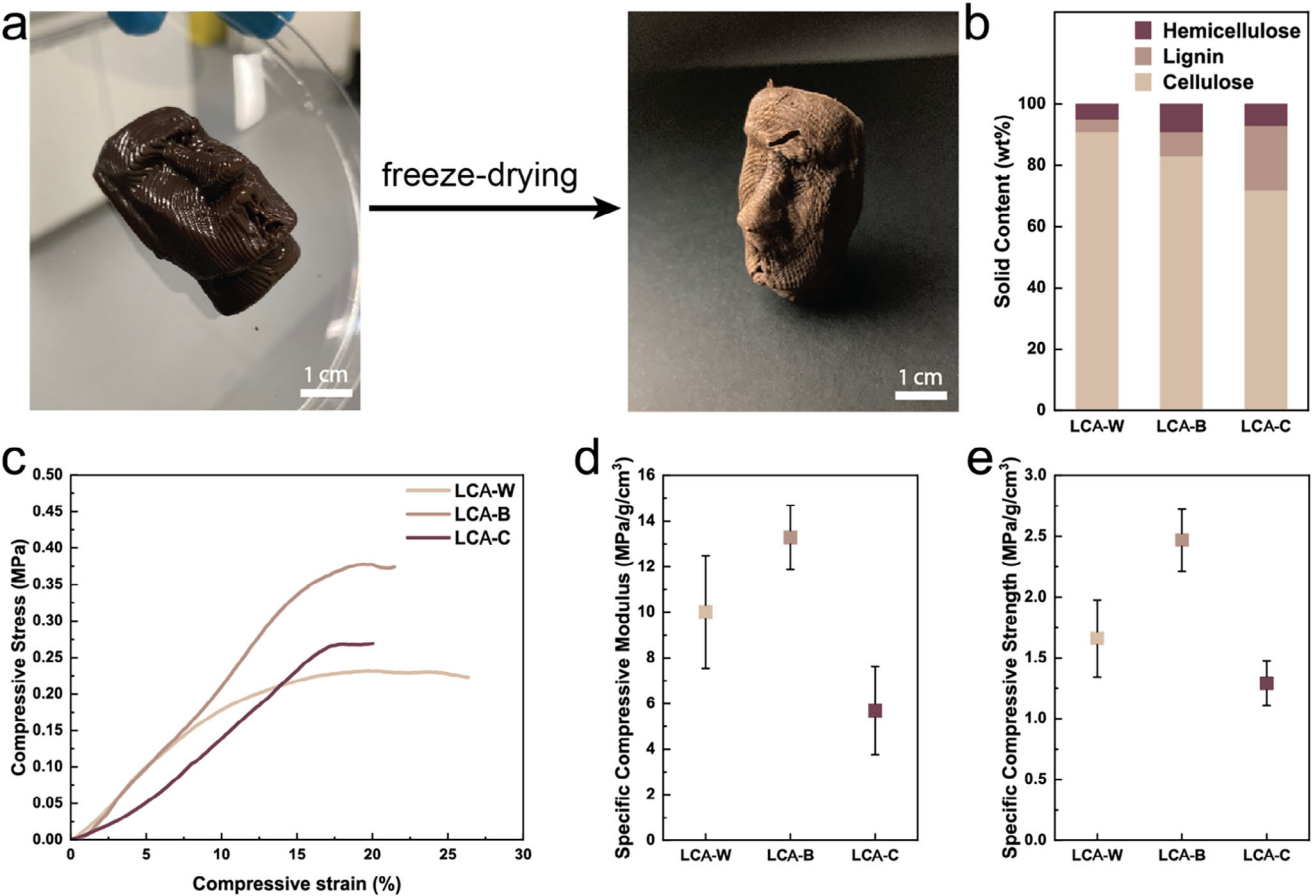
Mechanical characterization of LCAs. a) Photographs of the freeze‐drying process. The 3D printed LCP‐C structure is flash‐frozen in LN_2_ and lyophilized to produce a free‐standing LCA‐C. b) Compositional analysis of LCPs on a dry mass basis. c) Uniaxial compression tests of LCAs. d) Specific compressive moduli of LCAs as a function of lignin content. e) Specific compressive strength of LCAs as a function of lignin content.

**Figure 6:**
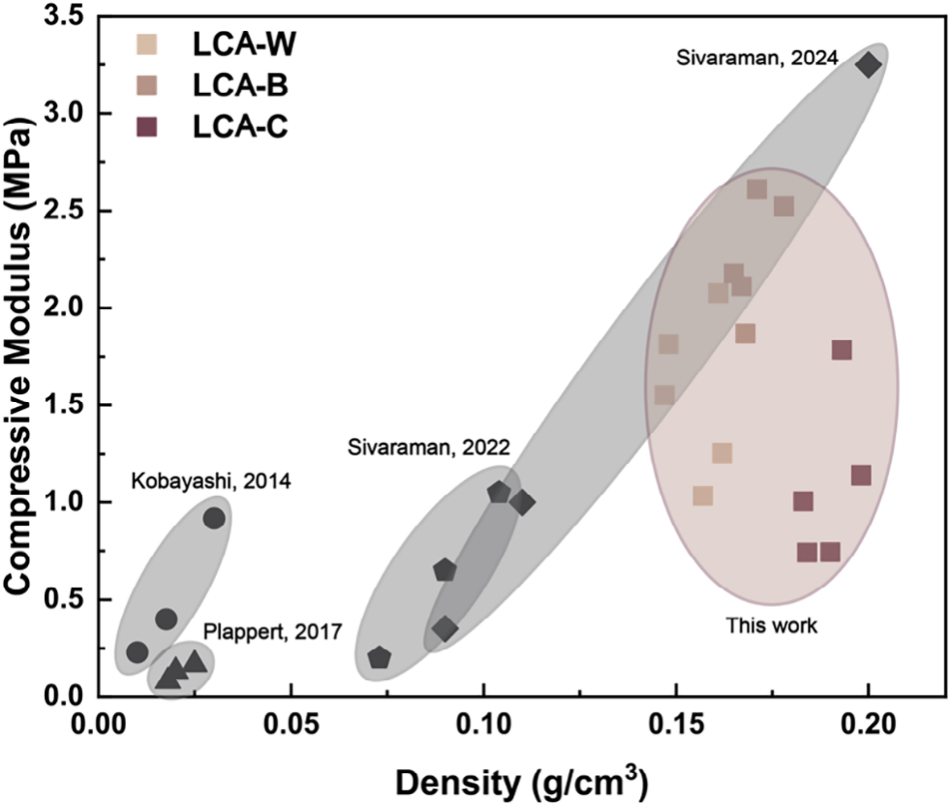
Ashby plot of cellulose‐based aerogels.

Since the 3D‐printed structures are free‐standing and composed of 14 wt.% cellulose, it is possible to freeze‐dry them, thus obtaining a lightweight aerogel, referred to as a lignocellulosic aerogel (LCA). The process and the relative composition of the LCAs are summarized in **Figure** **5**a,b, respectively. Briefly, the 3D printed construct was flash frozen at ‐196 °C in liquid nitrogen and then lyophilized at ‐20 °C and 0.01 mbar until the sample was completely dry. The freeze‐drying process suppresses capillary tension that would otherwise cause structural collapse during drying, thereby maintaining the original 3D‐printed shape and internal structure. The resulting aerogels are free‐standing and load‐bearing with densities ranging from 0.16 g cm^−3^ for LCA‐W to 0.20 g cm^−3^ for LCA‐C, as shown in Figure S10a (Supporting Information). Uniaxial compressive tests were performed to evaluate the mechanical performance of our LCAs, as shown in Figure 5c. Surprisingly, LCA‐B displays a compressive modulus that is almost 3‐fold higher than that measured for LCA‐C, as shown in Figure 5d and Figure S10b (Supporting Information). Similarly, the compressive strength of LCA‐B increases almost 2‐fold compared to that of LCA‐C, as shown in Figure 5e and Figure S10c (Supporting Information). We observe a strengthening effect when going from LCA‐W to LCA‐B, which have lignin contents of 4 and 8 wt.%, respectively, but a decrease in mechanical properties in LCA‐C, which has a lignin content of 20 wt.%. The decrease in mechanical performance beyond a specific lignin concentration is tentatively attributed to the formation of lignin‐rich domains within the heterogeneous LCP structure due to lignin's intrinsic hydrophobic characteristics. These lignin clusters may prevent the efficient percolation of hemicellulose or nanocellulose and localize weak points within the structure. Consequently, the final LCA becomes less structurally cohesive and weaker than its counterparts. To benchmark our LCAs against the state of the art of cellulose aerogels, we compared our mechanical performance to previously published reports in an Ashby plot in **Figure** **6**.^[^ ^41^, ^52^, ^53^, ^54^, ^55^ ^]^ From the Ashby plot, the LCAs display compressive moduli comparable to those of cellulose aerogels and native cork, thus further supporting the potential of 3D printed cellulose aerogels obtained from mildly processed LCPs.^[^ ^53^ ^]^

**Figure 7:**
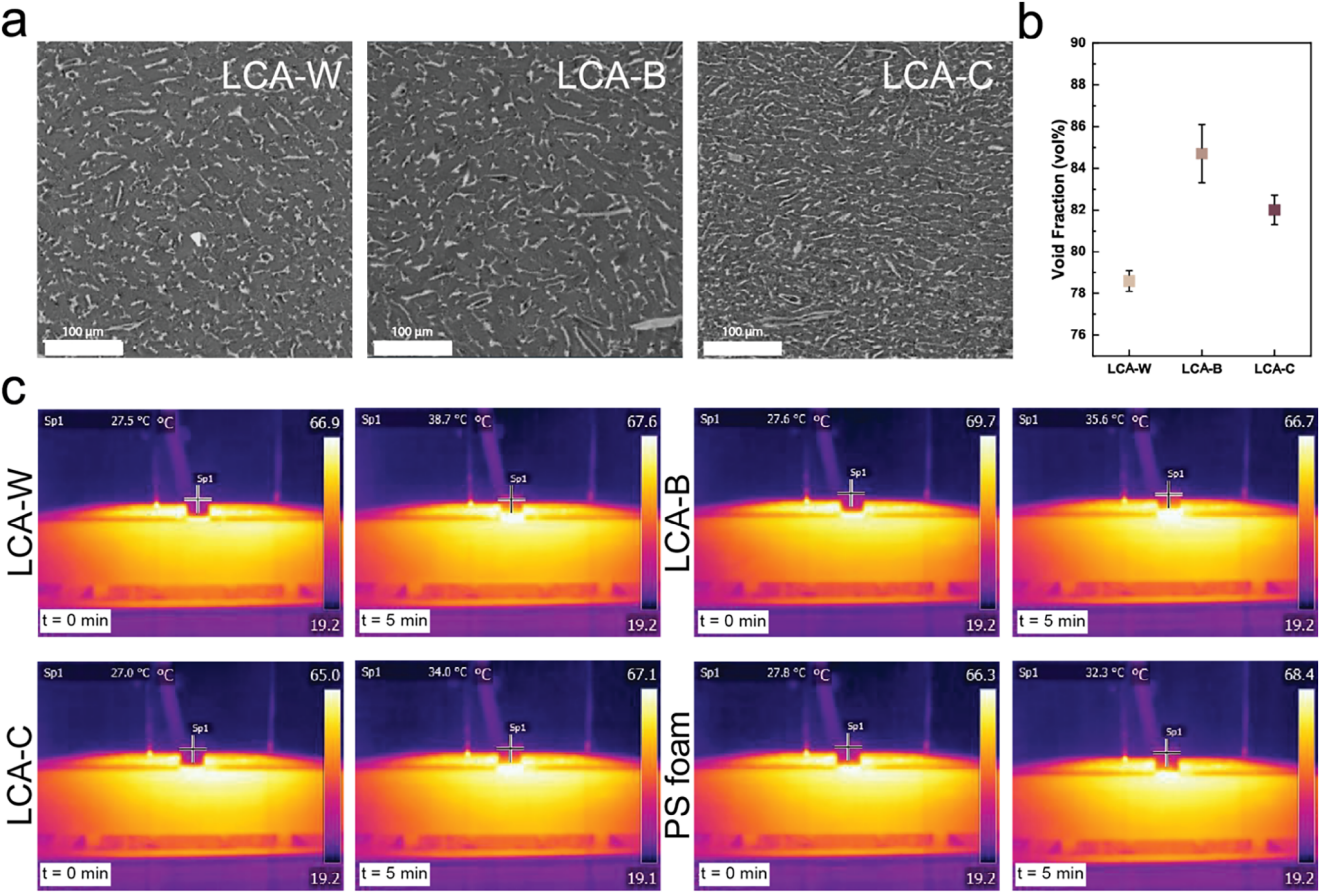
Thermal insulation properties of LCAs. a) µCT images of LCAs as a function of their lignin content. The images reveal a highly porous internal structure of the aerogels. b) Void fraction of LCAs as a function of lignin content. c) Thermal images of LCAs and PS foams as a function of time and lignin content. All LCAs display a relatively high thermal insulating property, comparable to that of commercially available PS foams.

A remarkable feature of cellulose aerogels is their extremely low density, light weight, and high degree of porosity.^[^ ^41^ ^]^ As a result, they can be employed in thermal insulation as sustainable alternatives to conventional synthetic materials, such as polystyrene (PS) foams. To verify if this is also the case for LCAs, micro‐computed tomography (µCT) was performed on our materials to evaluate their internal porosity, as shown in **Figure** **7**a and Figure S11 (Supporting Information). All LCAs display a high degree of porosity regardless of the lignin content. The corresponding void fraction reaches values as high as 85 vol% for LCA‐B, as shown in Figure 7b. The slightly higher void fraction measured in LCA‐B compared to LCA‐W and LCA‐C further supports the presence of an optimum lignin concentration that enhances LC percolation within the structure and thus prevents its internal collapse during freeze‐drying. Additionally, the ability of our constructs to dissipate heat was tested by placing the LCAs on a hotplate (70 °C) and monitoring the temperature change as a function of time, as shown in Figure 7c. The thermal images show an initial temperature of ≈27.5 °C across the different samples. After 5 min, LCA‐C shows a temperature increase of 7 °C, a value 60% lower than its LCA‐W counterpart. This substantial difference in thermal dissipation is attributed to a synergistic combination of high internal porosity and high lignin content. Indeed, the increase in lignin content is paired with a color shift of the LCA appearance from white to dark brown. While dark‐colored materials heat up faster than light colored ones, they also emit radiation faster, thus dissipating more heat.^[^ ^56^, ^57^ ^]^ Furthermore, LCA‐C displays a temperature increase that is 50% more than conventional polystyrene (PS) foams, thus making our material a promising candidate for sustainable insulating foams.

## 3 Conclusion

The production of our lignocellulosic aerogels involves three primary stages: material sourcing, material processing, and structure conversion. Scalability and limitations of each stage can be evaluated individually.

Regarding the scalability of material sourcing, the process utilizes the Sunburst method, an environmentally friendly extraction technique that converts waste hardwood chips (Birch) into lignocellulosic pastes (LCPs) at pilot scale, with plans for industrial‐scale production in the future. This method maintains the natural composition of wood, resulting in higher yields and reduced waste.^[^ ^45^ ^]^ The scalability of the processing step requires scaling up additive manufacturing processes, particularly through the use of multi‐nozzle printing systems. Literature highlights this approach's capability for high‐throughput production and the creation of complex structures with high resolution and shape retention.^[^ ^58^, ^59^ ^]^ Ultimately, to convert our LCPs into LCAs, the process faces inherent challenges associated with freeze‐drying, including capacity constraints, time consumption, and energy demands. However, advancements in bulk freeze‐drying techniques show potential for process scaling.^[^ ^60^ ^]^ Furthermore, research indicates the possibility of modifying raw material composition or chemistry to facilitate air‐drying, providing a less energy‐intensive option for large‐scale production.^[^ ^61^, ^62^ ^]^

In conclusion, we successfully demonstrated the fabrication of lignocellulosic aerogels from minimally processed pastes of varying lignin composition. Unlike the classic deconstruction‐reconstruction paradigm that underpins the state of the art in cellulose nanomaterial research, it is our minimal approach that leads to preservation of the compositional and structural motifs of natural wood sources, which in turn yields the unique properties of our materials that enable their additive manufacture, without additives or formulation. Thus, the unique rheological properties of LCPs result from their heterogeneous composition and the interplay between the microcrystalline cellulose particles and the thixotropic nano‐fraction component. These features enable the additive manufacturing of 3‐dimensional structures with high shape fidelity. Additionally, the relatively high solid content of the ink compared to conventional cellulose pastes allows the conversion of the 3D printed LCP structures into lightweight and load‐bearing LCAs. The final constructs have mechanical properties similar to cork and possess high thermal insulating properties. In summary, these features expand the application of less processed cellulose materials and offer a promising avenue for sustainable construction insulating materials, addressing the environmental challenges faced by conventional solutions.

## 4 Experimental Section

### Materials

Three different lignocellulosic pastes (white, blonde, and crude) were obtained from Fibenol OÜ. From the producer data sheet, LCP‐W has a solid content (SC) of 18.2 wt.%, pH of 10.4, degree of polymerization (DP) of 134, and a crystallinity index (CI) of 54.9%. LCP‐B has a SC of 17.2 wt.%, pH of 9.7, DP of 134, and CI of 55.8%. LCP‐C has a SC of 19.7 wt.%, pH of 11.7, DP of 134, and CI of 47.5%. Unless otherwise stated, all LCPs were diluted in Milli‐Q water to obtain a MCC solid content of 14 wt.% prior to further use. Microcrystalline cellulose (435 236), kraft lignin (370 959), and polyallylamine hydrochloride (PAH, 283 215) were purchased from Sigma‐Aldrich and were all used as received.

### Polarized Optical Microscopy (POM) of Lignocellulosic Pastes

POM measurements were performed on a Nikon Inverted Microscope with crossed‐polarizers and a lambda plate inserted to evaluate the microcrystalline component in the paste. Each sample was imaged, and the crystal size distribution was extracted through an image processing software (ImageJ). For each measurement, the largest diameter of the MCC (d_//_), the smallest diameter orthogonal to it (d_⟂_), and the aspect ratio (d_//_/d_⟂_) were collected. Results were calculated from at least 100 birefringent elements.

### Atomic Force Microscopy (AFM) of Lignocellulosic Nano‐Fraction

AFM measurements were conducted on the nano‐fraction component of the lignocellulosic pastes in air in tapping mode using a Cypher VRS (Oxford Instruments) with a silicon cantilever (AC200 TS, f = 100.4 – 169.43 kHz, F = 2.88 – 12.79 N/m). The nano‐fraction component was isolated by diluting the lignocellulosic pastes to 0.01 wt.% and centrifuging at 1000 *g* for 15 min. After the centrifugation, the supernatant was cast onto silicon substrates following a previously published protocol.^[^ ^63^ ^]^ Briefly, sample substrates were prepared by first dicing a silicon wafer and then cleaning it using an acidic piranha solution. The substrates were subsequently rinsed thoroughly with Milli‐Q water and dried under a nitrogen flow. To prevent the aggregation of the nano‐fraction during film drying, a cationic adhesive layer was first spin‐coated onto the wafer using a 0.1 wt.% PAH at 3000 rpm for 30 s, followed by rinsing with MilliQ water.

### Rheology of Lignocellulosic Pastes

Rheology measurements were performed on a DHR‐3 TA Instrument with an 8 mm diameter parallel plate steel geometry. All measurements were performed at 25 °C, with an 800 µm gap. The samples were allowed to relax for 200 s before each measurement. Frequency‐dependent viscosity measurements were performed at 0.5% strain. Amplitude sweeps were performed at 1.0 rad s^−1^ oscillation. Self‐healing measurements were performed at 1.0 rad s^−1^, alternating 200 s at 1% strain, with 200 s at 50% strain.

### 3D printing of Lignocellulosic Pastes

The lignocellulosic paste was loaded in a 3 mL Luer lock syringe. The syringe was sealed and centrifuged at 4500 rpm for 5 min to remove trapped air bubbles that would affect the printing quality. 3D printing was performed with a commercial 3D bioprinter (BIO X, Cellink) at 5 mm s^−1^ and 55 kPa, unless stated differently. The granular ink was extruded from a conical nozzle (22 G) through a pressure‐driven piston.

### Mechanical Characterization of Lignocellulosic Aerogels

Mechanical measurements were performed with a commercial machine (zwickiLine 5 kN, 50 N load cell, Zwick Roell). Compression tests were performed on cylindrical samples (d = 8 mm, h = 8 mm), compressed at a constant velocity of 3 mm min^−1^ until fracture or 80% strain was reached. The compressive modulus was calculated as the slope of the region from 0% to 5% strain.

### µCT Imaging and 3D Reconstruction of Lignocellulosic Aerogels

X‐Ray µCT was performed with an Ultratom micro tomography system (RX‐SOLUTIONS). The sample was scanned at a voxel resolution of 1.05 mm, with a voltage of 45 kV and a current of 166 mA. Amira‐Avizo v.2019.4 software was used for reconstruction, segmentation, and visualization.

### Thermal Insulation of Lignocellulosic Aerogels

Thermal insulation measurements were performed on a hot plate at 70 °C and recorded with a thermal camera (FLIR C5) with an emissivity value of 0.95. Thermal photographs were acquired with a time interval of 5 min to qualitatively assess the ability of the LC aerogels and PS foam to dissipate heat.

## Conflict of Interest

The authors declare no conflict of interest.

## Supporting information



Supporting Information

Supplemental Movie 1

## Data Availability

The data that support the findings of this study are available from the corresponding author upon reasonable request.
