# Supplementary material for: Additive Manufacturing of Lignocellulosic Aerogels from Minimally Processed Waste Streams
Source: Small. 2025 Jul 24;21(37):e12509. doi: 10.1002/smll.202412509 (PMC12444824; doi:10.1002/smll.202412509)
Supplement: Supplementary file 1 — Supporting Information [file SMLL-21-e12509-s001.docx]

Supporting Information

**Additive Manufacturing of Lignocellulosic Aerogels from Minimally Processed Waste Streams**

Matteo Hirsch,^1^ Kosuke Ayama,^1^ Gert Preegel,^2^ and Tiffany Abitbol^1,*^

^1^Sustainable Materials Laboratory, Institute of Materials, École Polytechnique Fédérale de Lausanne, 1015 Lausanne, Switzerland

^2^Fibenol. Tallinn, Estonia

*Email: tiffany.abitbol@epfl.ch

**Supplementary Movie M1.** Video of a 3D printed LCP-W grid.


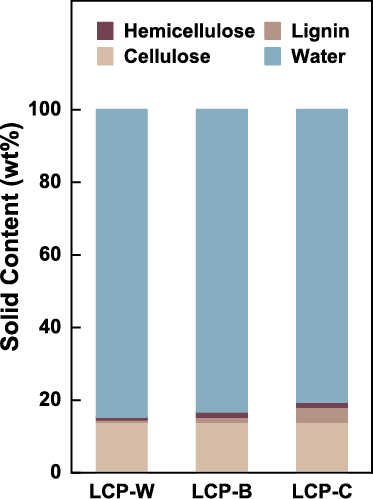


**Figure S1 | Composition of the LCPs.** Solid content of different lignocellulosic pastes.


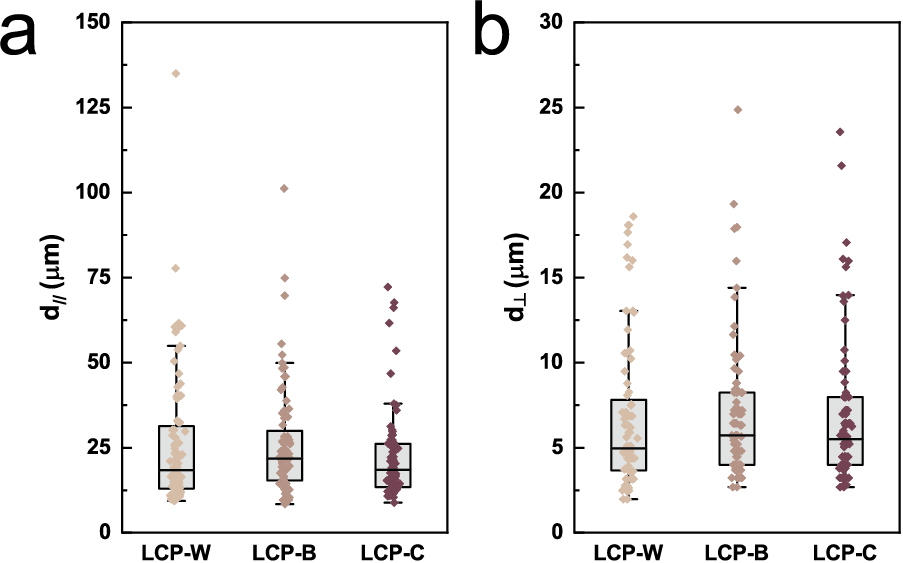


**Figure S2 | Size distribution of MCC in LCPs. a-b**, MCC size distribution as a function of LCP composition.

**
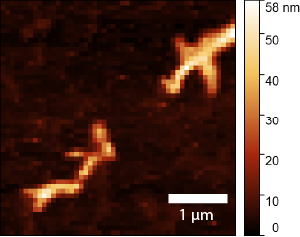
**

**Figure S3 | AFM of LCP-B.** AFM images of LCP-B nano-fraction obtained by centrifugation the paste at 0.01 wt%. LCP-B shows rod-shaped aggregates and smaller nanoparticles attributed to lignin.


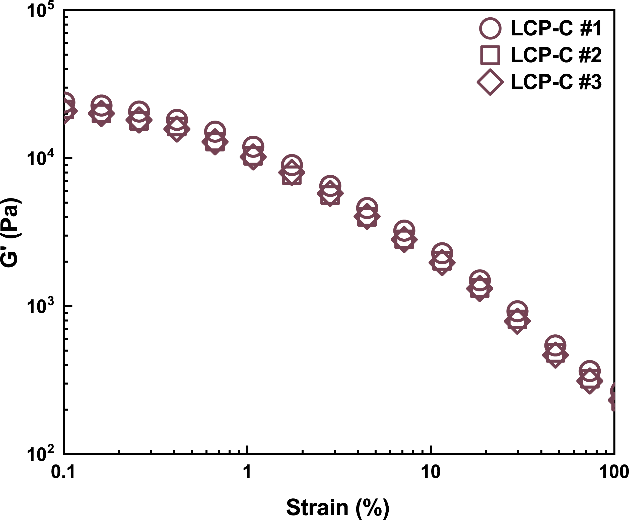


**Figure S4 | Rheology Repeats.** Overlap of three independent measurements of LCP-C 14wt%. The plot displays very similar rheological behavior for each of the individual measurements.

**
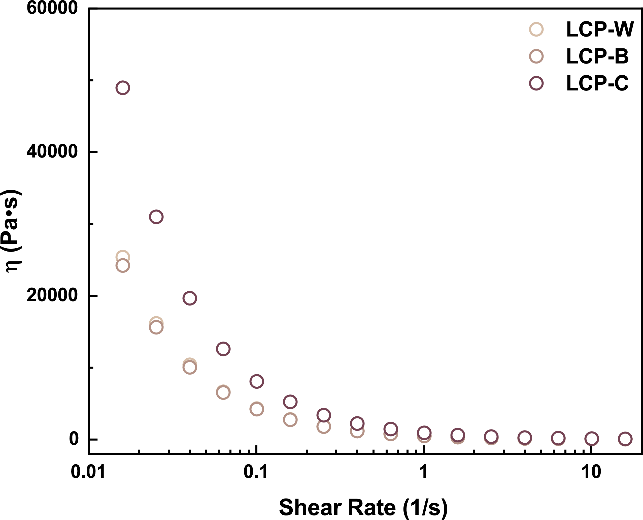
**

**Figure S5 | Shear Thinning Magnified.** Shear thinning curves from Figure 3b presented in a semi-logarithmic scale to enhance visibility of the individual datasets.

**
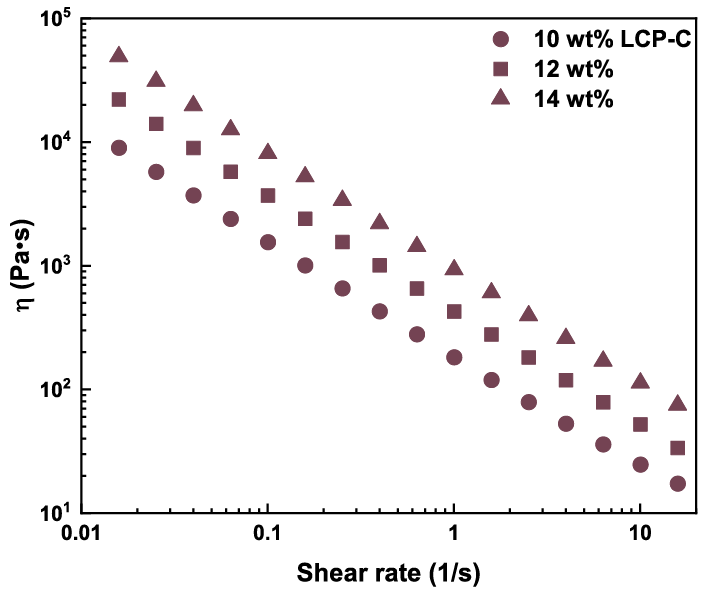
**

**Figure S6 | Rheology of LCP-C.** Frequency sweeps of LCP-C as a function of cellulose content.

**
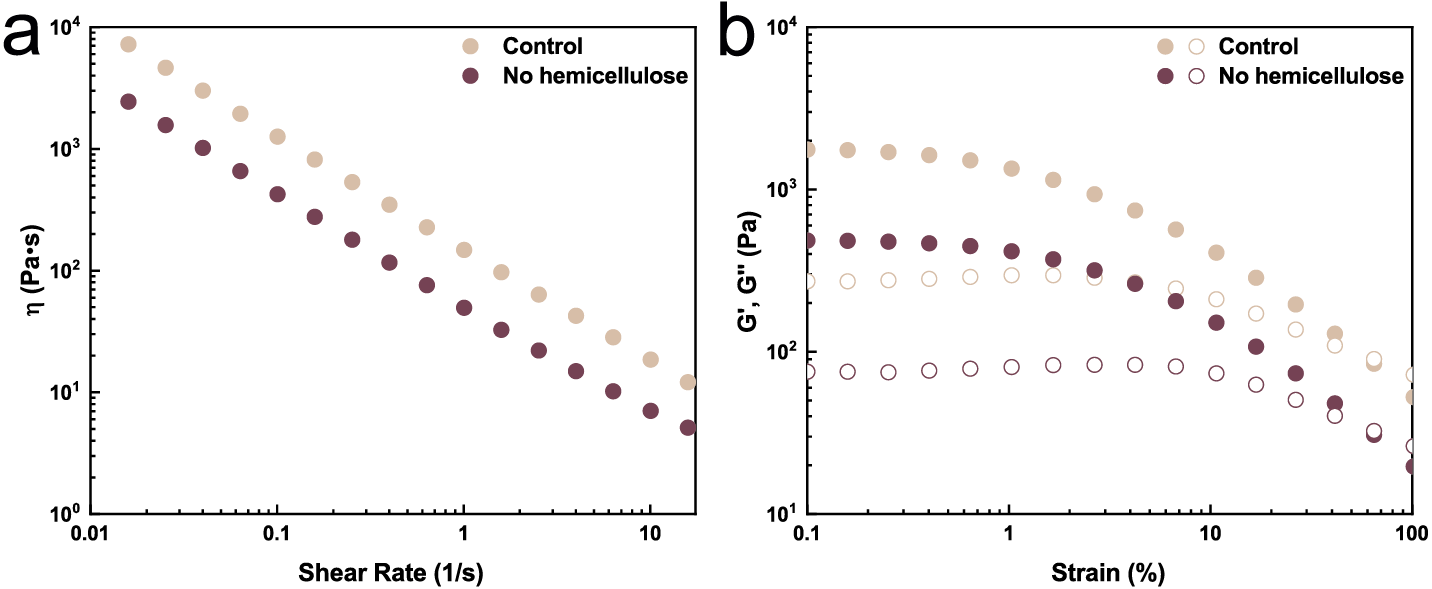
**

**Figure S7 | Rheology of LCP-W.** **a**, Frequency sweeps of LCP-W as a function of hemicellulose content. **b**, Amplitude sweeps of LCP-W as a function of hemicellulose content.

**
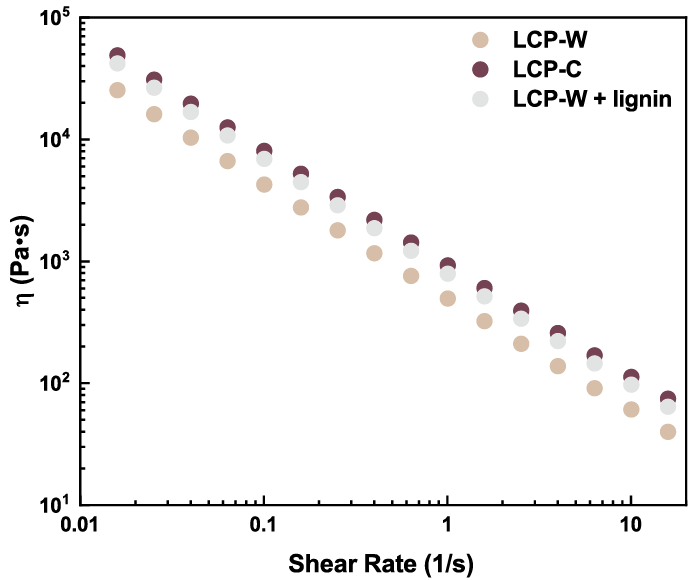
**

**Figure S8 | Rheology of pre-mixed LCPs.** Frequency sweeps of pre-mixed LCP-W + lignin, as-extracted LCP-W, and LCP-C.

**
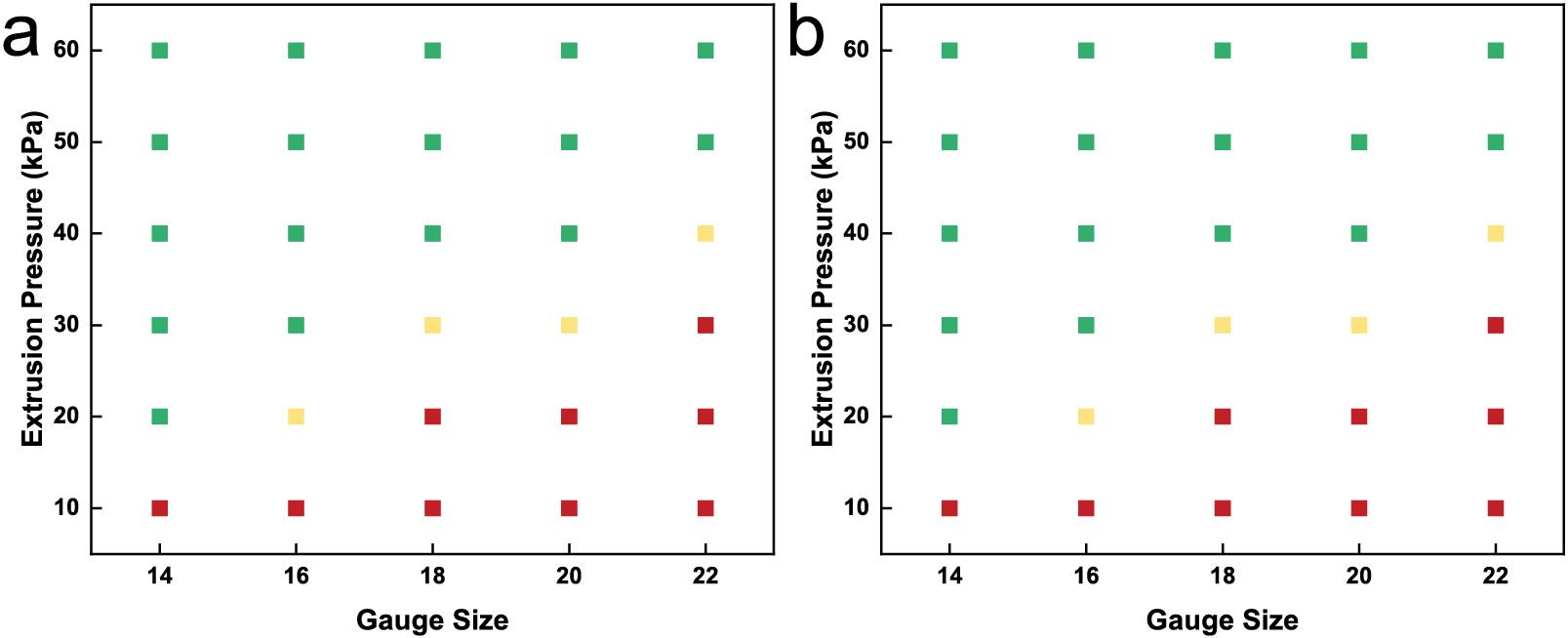
**

**Figure S9 | Printing window of LCPs.** Printing window of (**a**) LCP-W and (**b**) LCP-B as a function of nozzle size and extrusion pressure. Red points correspond to no printing, yellow to discontinuous printing at 5 mm/s, and green to continuous printing at 5 mm/s.

**
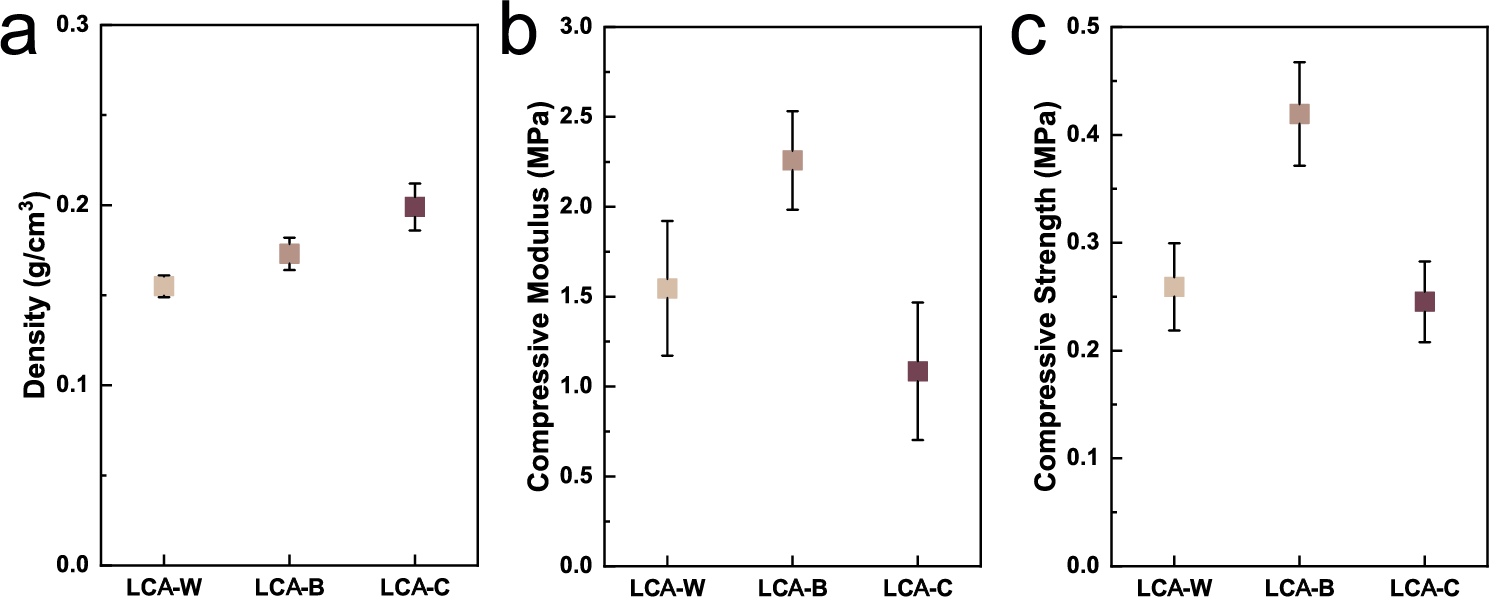
**

**Figure S10 | Mechanical characterization of LCFs. a**, Density measurements of LCFs as a function of lignin content. **b**, Compressive modulus of LCFs as a function of lignin content. **c**, Compressive strength of LCFs as a function of lignin content.

**
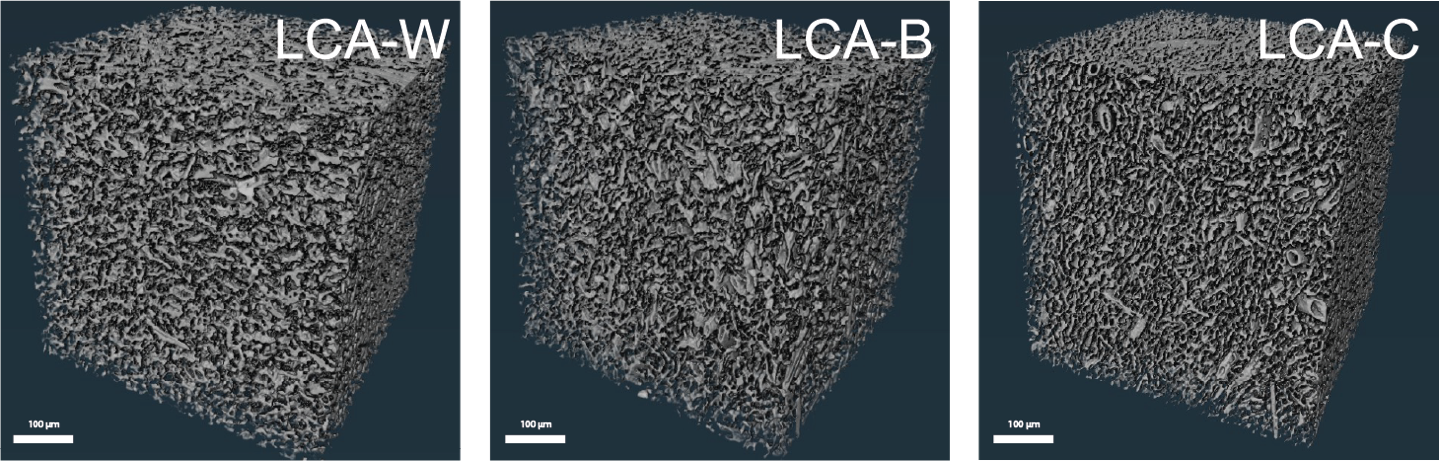
**

**Figure S11 | µCT of LCFs.** 3D reconstruction of LCF-W, LCF-B, and LCF-C. The tomographic reconstruction reveals a highly porous network regardless of the lignin content.
